# An epithelial marker promoter induction screen identifies histone deacetylase inhibitors to restore epithelial differentiation and abolishes anchorage independence growth in cancers

**DOI:** 10.1038/cddiscovery.2016.41

**Published:** 2016-06-13

**Authors:** H M Tang, K T Kuay, P F Koh, M Asad, T Z Tan, V Y Chung, S C Lee, J P Thiery, RY-J Huang

**Affiliations:** 1 Cancer Science Institute of Singapore, National University of Singapore, Centre for Translational Medicine NUS Yong Loo Lin School of Medicine, Singapore 117599, Singapore; 2 Department of Obstetrics & Gynaecology, National University Health System, Singapore 119228, Singapore; 3 National University Cancer Institute of Singapore, National University Health System, Singapore 119228, Singapore; 4 Comprehensive Cancer Center, Institut Gustave Roussy, 114 Rue Edouard Vaillant, 94805 Villejuif, France; 5 CNRS UMR 7057, Matter and Complex Systems, Université Paris Diderot, 5 rue Thomas-Mann, 75013 Paris, France; 6 Department of Biochemistry, National University of Singapore, Singapore 117597, Singapore; 7 Department of Anatomy, Yong Loo Lin School of Medicine, National University of Singapore, Singapore 117597, Singapore

## Abstract

Epithelial–mesenchymal transition (EMT), a crucial mechanism in development, mediates aggressiveness during carcinoma progression and therapeutic refractoriness. The reversibility of EMT makes it an attractive strategy in designing novel therapeutic approaches. Therefore, drug discovery pipelines for EMT reversal are in need to discover emerging classes of compounds. Here, we outline a pre-clinical drug screening platform for EMT reversal that consists of three phases of drug discovery and validation. From the Phase 1 epithelial marker promoter induction (EpI) screen on a library consisting of compounds being approved by Food and Drug Administration (FDA), Vorinostat (SAHA), a histone deacetylase inhibitor (HDACi), is identified to exert EMT reversal effects by restoring the expression of an epithelial marker, E-cadherin. An expanded screen on 41 HDACi further identifies 28 compounds, such as class I-specific HDACi Mocetinosat, Entinostat and CI994, to restore E-cadherin and ErbB3 expressions in ovarian, pancreatic and bladder carcinoma cells. Mocetinostat is the most potent HDACi to restore epithelial differentiation with the lowest concentration required for 50% induction of epithelial promoter activity (EpIC-50).The HDACi exerts paradoxical effects on EMT transcriptional factors such as *SNAI* and *ZEB* family and the effects are context-dependent in epithelial- and mesenchymal-like cells. *In vitro* functional studies further show that HDACi induced significant increase in anoikis and decrease in spheroid formation in ovarian and bladder carcinoma cells with mesenchymal features. This study demonstrates a robust drug screening pipeline for the discovery of compounds capable of restoring epithelial differentiation that lead to significant functional lethality.

## Introduction

Epithelial–mesenchymal transition (EMT) is a gradual process whereby epithelial cells lose their epithelial features, enter into the hybrid intermediate state while gaining some mesenchymal features, and ultimately transdifferentiate into mesenchymal cells.^1^ This process is reversible in nature with the hybrid intermediate cells being shown to be in the most plastic state.^2^ As EMT has been implicated as one of the contributing mechanisms to the aggressiveness of carcinoma during disease progression, cancer stemness and chemoresistance,^3^ the possibility to reverse the aggressiveness by reversing EMT and restoring the epithelial differentiation has emerged to be an appealing strategy in cancer treatment.^4^

The main molecular mechanism for EMT is mediated by several key transcription factors (TF) to regulate their downstream targets at the transcriptional, translational and post-translational levels that are associated with transdifferentiation.^5^ Upstream to this, several signaling pathways responding to the external cues are crucial to mediate the convergence of the signals to the main transcriptional EMT factors. Therefore, these signaling pathways such as transforming growth factor-*β* (TGF*β*), hepatocyte growth factor, insulin-like growth factor 1, epidermal growth factor (EGF), fibroblast growth factor and platelet-derived growth factor pathways have been suggested to be the desirable targets against EMT.^5,6^ EMT reversal and restoring epithelial differentiation has been shown to achieve by kinase inhibitors, such as the TGF*β* receptor types I and II inhibitor LY2109761,^7^ the Src-kinase inhibitor saracatinib (AZD0530)^8^ and the triple angiokinase inhibitor nintedanib (BIBF1120),^9^ which acts to upregulate E-cadherin expression both *in vitro* and *in vivo*. However, most of these kinase inhibitors have not been demonstrated to exhibit anti-EMT-related effects in the clinical settings except for nintedanib (BIBF1120) which has been shown to resolve idiopathic pulmonary fibrosis by inhibiting the TGF*β* pathway.^10^ Therefore, there is a need to explore other classes of compounds.

The concept of EMT reversal is similar to the differentiation therapy^11^ that involves re-programming of the cancer cells^12^ from the mesenchymal to epithelial trait. With EMT being a crucial differentiation-based developmental model in cancers, the identification of targetable pathways to re-program the mesenchymal trait would be very promising. Differentiation therapy with all-trans retinoic acid has been developed to treat acute myeloid leukemia.^13^ Over the years, several pharmaceuticals and natural compounds have also been shown to re-program the differentiation pathways in leukemia cells.^14^ Increasing evidences have shown that the differentiation therapy in solid tumors is possible.^15^ The cancer stem cell (CSC) concept have further provided the theoretical and practical grounds to develop the differentiation therapy in solid tumors such as breast and renal cancers.^11,16^ During the differentiation of stem cells, epigenetic regulations are the key governing mechanism and thus pose as an appealing therapeutic target for differentiation therapy in CSC.^17^ The implication of EMT and CSC^18^ thus makes epigenetic modifiers a promising class of compounds for EMT reversal and restoring epithelial differentiation.

In this study, we describe the discovery of histone deacetylase (HDAC) inhibitors from a 3-phase drug screening pipeline for restoring epithelial differentiation. We demonstrate that these HDAC inhibitors (HDACi) induce different effects in ovarian cancer cells with different EMT statuses. The EMT reversal effect of restoring E-cadherin ErbB3 expressions by HDACi is also validated in non-ovarian cancer cells such as pancreatic and bladder cancers. Restoration of epithelial differentiation by these HDACi has a functional relevance in overcoming anoikis resistance and anchorage independence growth.

## Results

### An epithelial marker promoter induction screen identifies EMT reversal agents

**Figure 1:**
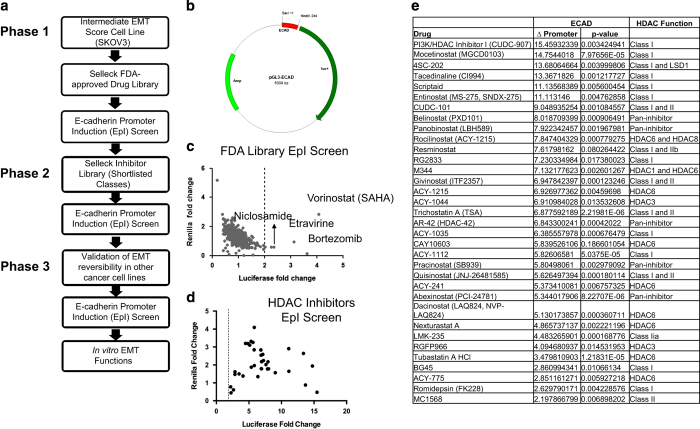
Drug library screens identify EMT reversal agents. (**a**) Flow chart of the pre-clinical drug discovery pipeline for EMT reversal. EpI screen uses E-cadherin (Ecad) promoter activity as the readout. (**b**) pGL3 luciferase plasmid, containing a short promoter region of Ecad of 233 bp (−108/+125). (**c**) Dot plot summarizes the results of Phase I EpI screen. *y*-Axis indicates cell viability and *x*-axis represents induction of Ecad promoter activity. Five drugs, Vorinostat, Bortezomib, Etravirine, Niclosamide and Crystal violet, were identified with more than twofold Ecad promoter activity. (**d**) Dot plot summarizes the results of Phase 2 screen on HDAC inhibitors. (**e**) Table summarizes the fold change of Ecad promoter activity induction by HDACi. *P*-value summary of change in Ecad promoter activity analysis using paired *t*-test for comparison between drug-treated and DMSO-treated groups.

The EMT reversal application is based on re-differentiating cancers along an EMT spectrum which is quantitatively defined by continuous EMT scores.^19^ Therefore, establishing a robust drug discovery pipeline based on the reversibility of EMT is mandatory. We have established a pre-clinical drug discovery pipeline (Figure 1a) for EMT reversal by using the re-expression of an epithelial differentiation marker, E-cadherin, as the readout. We have previously demonstrated that a short version of the E-cadherin promoter region containing the E-box sequences can be used to reflect an increase in *CDH1* promoter activity upon EMT reversal.^9^ The discovery pipeline starts from the Phase 1 screening of a Food and Drug Administration (FDA)-approved drug library in a human ovarian cancer cell line harboring an intermediate EMT score, SKOV3, transiently transfected with the pGL3 luciferase plasmid containing the short promoter region of E-cadherin (Figure 1b, Supplementary Materials). This screening platform is therefore referred as the epithelial marker promoter induction (EpI) screen. The Phase 2 screen expands to compounds belonging to the same class of action as the shortlisted compounds from Phase 1. These compounds would be subjected to another round of EpI screen in SKOV3. Compounds selected from Phase 2 are further tested in various cancer cell lines annotated with similar EMT scores to SKOV3 including, MDA-MB-231 (breast), T24 (bladder), A549 (lung) and Mia-Paca2 (pancreas). The dose-dependent dynamic ranges of the compounds to induce re-expression of E-cadherin are determined *in vitro*. Compounds with good linear dynamic ranges in re-expressing E-cadherin without apparent cytotoxicity will then be tested in the Phase 3 functional validation.

From the Phase 1 EpI screen, five compounds (Vorinostat, Bortezomib, Etravirine, Niclosamide and Crystal violet) were identified as potent EMT reversal agents with increased E-cadherin promoter activities (Figure 1c) in SKOV3. Among them, Vorinostat (SAHA) showed no apparent cytotoxicity represented by the Renilla readout at the screening concentration of 5 *μ*M. This was further validated by the MTS assay (Supplementary Figure 1). We subsequently expanded the screen to include other compounds belonging to the same class of action as Vorinostat, which is the histone deacetylase inhibitor (HDACi). Among 41 HDACi screened, 28 showed enhanced E-cadherin promoter activities of at least twofold (Figures 1d and e). Interestingly, in the order of fold changes of E-cadherin promoter activities, the top ranking compounds (CUDC-907, Mocetinostat, 4SC-202, CI994, Scriptaid and Entinostat) all have selectivity towards the class I HDAC (Figure 1e). In particular, two compounds with dual inhibition of HDAC and kinase activities, CUDC-907 and CUDC-101, also emerged from this screen. This is consistent with the findings that CUDC-101 prevents *in vitro* and *in vivo* aggressiveness via suppressing EMT.^20,21^ We previously reported that a triple angiokinase inhibitor, Nintedanib, could restore E-cadherin expression.^9^ Therefore, the class of angiogenesis inhibitors was also subjected to the Phase 1 EpI screen. Among 28 compounds screened, 10 showed enhanced E-cadherin promoter activities of at least twofold (Supplementary Figures 2A and B). Of note, these angiogenesis inhibitors were all less potent than the selective class I HDACi in EpI.

### Class I HDACi exhibits dose-dependent induction of E-cadherin and ErbB3 expression

**Figure 2:**
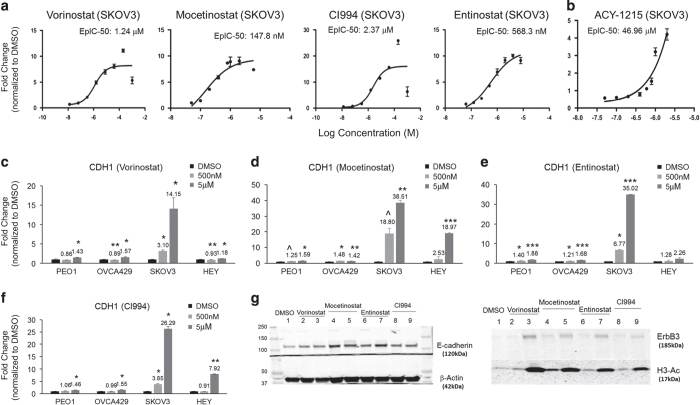
HDACi exhibits dose-dependent induction of E-cadherin gene expression. Induction dose–response curves indicate EpIC-50 of E-cad promoter in Vorinostat, Mocetinostat, Entinostat and CI994 (**a**), ACY-1215 (**b**) in SKOV3. *y*-Axis represents fold change of Ecad promoter activity induction and *x*-axis indicates various concentrations of the respective drug. EpIC-50 values were measured using Dual-Glo Luciferase Assay, and generated by curve fitting using three-parameter analysis. Graphs indicate changes in expression of *CDH1* after treating PEO1, OVCA429, SKOV3, and Hey with 500 nM (light gray bars), 5 *μ*M (dark gray bars) of Vorinostat (**c**), Mocetinostat (**d**), Etinostat (**e**), CI994 (**f**) and DMSO (black bars) for 24 h. Statistical significant at ^*P*<0.1, *P <0.05, **P<0.01, ***P<0.005 as compared with control (paired *t*-test). (**g**) Western blots showing E-cadherin and ErbB3 expressions in 500 nM (lane 2, 4, 6 and 8) and 5 *μ*M (lane 3, 5, 7 and 9) HDACi and DMSO (lane 1)-treated SKOV3 cells. *β*-Actin as the loading control and acetylated histone 3 (H3-Ac) as the histone acetylation control.

We have previously shown that the degree of EMT reversal could be represented by determining the concentration required for 50% induction of E-cadherin (E-cad) promoter activity.^9^ The EpIC-50 of Vorinostat, Mocetinostat, CI994 and Entinostat in SKOV3 was determined to be 1.24 *μ*M, 147.8 nM, 2.37 *μ*M and 568.3 nM, respectively (Figure 2a). A HDAC6-specific inhibitor, ACY-1215, though showing 7.8-fold of increase in EpI at the screening concentration of 5 *μ*M (Figure 1e), its dose–response curve only started to display linearity at much higher concentrations with an EpIC-50 reaching 47 *μ*M (Figure 2b). It is known that HDAC6-specific inhibitors like ACY-1215 would display cross selectivity to class I HDAC at high concentrations. Therefore, our data indicates that the EpI effect is specifically attributed to the inhibition of class I HDAC.

The linearity of EpIC was concordant with the dose dependency increase in transcript expressions of E-cadherin (*CDH1*) upon treating SKOV3 with 500 nM and 5 *μ*M of Vorinostat (Figure 2c). However, this trend was only evident in mesenchymal-like but not in epithelial-like cell lines. By using four ovarian cancer cell lines representing different EMT phenotypes,^8^ PEO1 (E), OVCA429 (IE), SKOV3 (IM), Hey (M), the induction of *CDH1* expression by Mocetinostat, Entinostat and CI994 was only greatly enhanced in the mesenchymal lines SKOV3 and Hey (Figures 2d–f). Mocetinostat appeared to be a very potent HDACi in the induction of *CDH1* expression. Mocetinostat-enhanced *CDH*1 expressions up to 18.8- and 38.5-fold at 500 nM and 500 *μ*M in SKOV3; 2.53- and 18.97-fold at 500 nM and 500 mM in Hey (Figure 2d). Entinostat enhanced *CDH1* expressions only in SKOV3 with 6.77- and 35.0-fold at 500 nM and 5 *μ*M but not in Hey (Figure 2e). CI994 enhanced *CDH1* expressions only in SKOV3 with 3.85- and 26.29-fold at 500 nM and 5 *μ*M and 7.92-fold at 5 *μ*M in Hey (Figure 2f). The E-cadherin protein expressions were also induced (Figure 2g). All three HDACi induced minimal increase of *CDH1* expressions in the epithelial lines PEO1 and OVCA429 below twofold, suggesting that the regulation of *CDH1* transcript level might have reached equilibrium in these epithelial-like lines.

**Figure 3:**
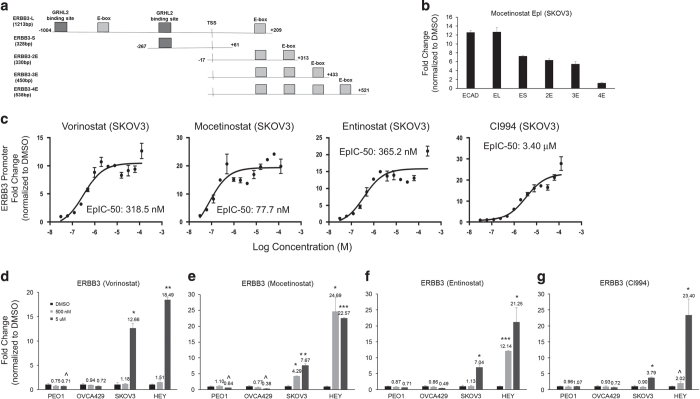
HDACi induces *ERBB3* promoter activity and ErbB3 gene expression. (**a**) Scheme showing various clones of *ERBB3* promoter with different sequences relative to the transcription start site. (**b**) Fold change of promoter activity (*y*-axis) in response to Mocetinostat in different promoter clones (*x*-axis). (**c**) Induction dose–response curves indicate EpIC-50 of *ERBB3* promoter in Vorinostat, Mocetinostat, Entinostat and CI994 in SKOV3. *y*-Axis represents fold change of *ERBB3* promoter activity induction and *x*-axis indicates various concentrations of the respective drug. Graphs indicate changes in expression of *ERBB3* after treating PEO1, OVCA429, SKOV3 and Hey with 500 nM (light gray bars), 5 *μ*M (dark gray bars) of Vorinostat (**d**), Mocetinostat (**e**), Etinostat (**f**), CI994 (**g**) and DMSO (black bars) for 24 h. Error bars represented S.E.M. from triplicate cultures. Statistical significant at ^*P*<0.1, *P<0.05, **P<0.01 as compared with control (paired *t*-test).

We further tested whether the EpI effect could be generalized to other epithelial genes. Another epithelial-specific gene, *ERBB3*, a direct transcriptional target of the epithelial gatekeeper GRHL2,^22^ has emerged as a promising therapeutic target in cancer. We generated various clones of the *ERBB3* promoter to cover two GRHL2-binding sites and five E-box sites close to the transcription start site of *ERBB3* (Figure 3a). We utilized Mocetinostat to test which promoter clones would show similar activity as the E-cad promoter. *ERBB3*-L, the clone of 1.2-kb sequence spanning the two upstream GRHL2-binding sites, one upstream E-box site and one downstream E-box site to transcription start site showed similar fold of increase of promoter activity to E-cad (Figure 3b). The *ERBB3*-L promoter activities also showed a dose-dependent induction following HDACi treatments. The EpIC-50 concentrations based on *ERBB3* promoter activities in SKOV3 were determined to be 318.5 nM, 77.7 nM, 365.2 nM and 3.40 *μ*M for Vorinostat, Mocetinostat, Entinostat and CI994, respectively (Figure 3c), and were consistently lower than those for E-cad promoter. Similar to the results of E-cad promoter activities, Mocetinostat showed the lowest EpIC-50 for the *ERBB3* promoter. This suggested that epithelial genes might have different thresholds for epigenetic regulations to induce transcriptions. Similar to *CDH1*, *ERBB3* expressions were significantly increased by these HDACi in the mesenchymal lines, with Mocetinostat being the most potent HDACi (Figures 3d–g and 2g). Collectively, our data suggested that by using epithelial gene promoters as readouts, the restoration of epithelial differentiation can be achieved by the EpI screen strategy.

### Cancer cells with similar EMT status show comparable EpI responses induced by class I HDACi

**Figure 4:**
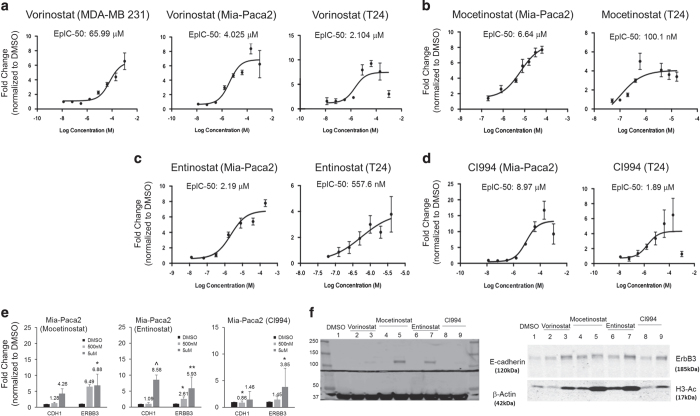
Different cancer cell types show varied EpI responses induced by class I HDACi. (**a**) EpI dose–response curve indicates Ecad promoter activity in fold change (*y*-axis) in MDA-MB-231, MIA-PaCa2 and T24 after various concentrations of Vorinostat treatment (*x*-axis). EpI dose–response curve represents Ecad promoter activity in fold change (*y*-axis) in MIA-PaCa2 and T24 after various concentrations of Mocetinostat (**b**), Entinostat (**c**) and CI994 (**d**) treatment (*x*-axis). EpIC-50 values were measured using Dual-Glo Luciferase Assay, and generated by curve fitting using three-parameter analysis. (**e**) Graph shows fold changes (*y*-axis) in *CDH1* and *ERBB3* expressions (*x*-axis) after treating Mia-Paca2 with 500 nM (light gray bars) and 5 *μ*M (dark gray bars) of Mocetinostat, Entinostat and DMSO (black bars) for 24 h. Statistical significant at ^*P*<0.1, *P<0.05, **P<0.01 as compared with control (paired *t*-test). (**f**) Western blots showing E-cadherin and ErbB3 expressions in 500 nM (lane 2, 4, 6 and 8) and 5 *μ*M (lane 3, 5, 7 and 9) HDACi and DMSO (lane 1) treated T24 cells. *β*-Actin as the loading control and acetylated histone 3 (H3-Ac) as the histone acetylation control.

We next explored whether the EpI screen could be applied to cancer cells other than ovarian cancer. Cancer cell lines with similar EMT scores^19^ as SKOV3 (+0.28), MDA-MB-213 (+0.391), T24 (+0.356) and Mia-Paca2 (+0.4345), were selected to validate the EMT reversal effect by HDACi. The EpIC-50 analysis of Vorinostat showed that MDA-MB-231 had the highest EpIC-50 up to ~66 *μ*M, compared with 4.0 *μ*M in Mia-Paca2 and 2.1 *μ*M in T24 (Figure 4a). This suggested that MDA-MB-231 might have higher barrier to restore *CDH1* expression by HDACi. The bladder carcinoma cell line, T24, showed very similar EpIC-50 ranges comparable to SKOV3 at 100.1 nM for Mocetinostat, 557.6 nM for Entinostat, and 1.89 *μ*M for CI994 (Figures 4b–d). The pancreatic carcinoma cell line, Mia-Paca2, showed a micro-molar range of EpIC-50 for all HDACi at 6.64, 2.19 and 8.97 *μ*M for Mocetinostat, Entinostat and CI994, respectively (Figures 4b–d). Similar to the ovarian lines, the EpI responses were also correlated with the induction of *CDH1* and *ERBB3* transcriptions (Figure 4e), as well as E-cadherin and ErbB3 protein expressions (Figure 4f) in Mia-Paca2 and T24 cells. Cancer cells with similar profiles as SKOV3, Mia-Paca2 and T24 might be the better responders for class I HDACi-induced EMT reversal. However, given that MDA-MB-231 did not showed EpI responses, the restoration of *CDH1* and *ERBB3* expression in mesenchymal-like cells by HDACi is therefore still context dependent. Collectively, our data suggested that the EpI screen strategy is an efficient and robust method to identify targets for restoring the epithelial differentiation.

### Different effects of class I HDACi on EMT TFs in epithelial- and mesenchymal-like cells

**Figure 5:**
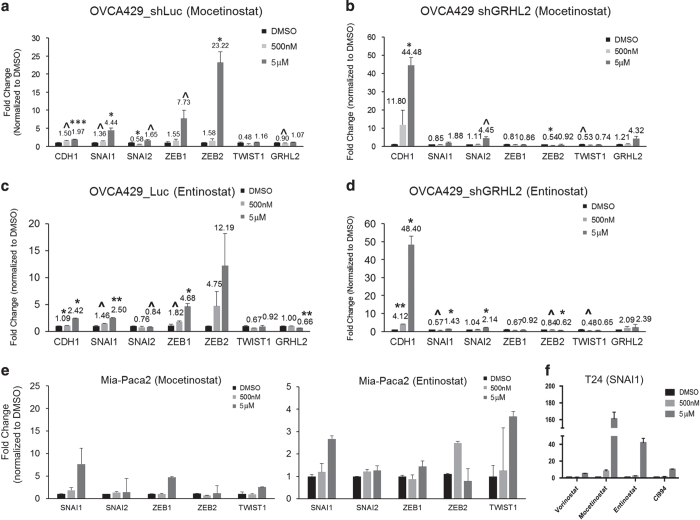
EMT reversal induced by class I HDACi is only evident in mesenchymal-like cell lines. Graph shows fold changes (*y*-axis) in *CDH1* and EMT TFs, *SNAI1*, *SNAI2*, *ZEB1* and *ZEB2*, expressions after treating OVCA429_shLuc with 500 nM (light gray bars) and 5 *μ*M (dark gray bars) of Mocetinostat (**a**), Entinostat (**c**) and DMSO (black bars) for 24 h. Graph shows fold changes (*y*-axis) in *CDH1* and EMT TFs expressions, after treating OVCA429_shGHL2 with 500 nM (light gray bars) and 5 *μ*M (dark gray bars) of Mocetinostat (**b**), Entinostat (**d**) and DMSO (black bars) for 24 h. Error bars represented S.E.M from triplicate cultures. Statistical significant at ^*P*<0.1, *P <0.05, **P<0.005 as compared with control (paired *t*-test). (**e**) Graphs show fold changes (*y*-axis) in EMT TFs expressions, after treating Mia-Paca2 with 500 nM (light gray bars) and 5 *μ*M (dark gray bars) of Mocetinostat and Entinostat. (**f**) Graph shows the SNAI1 expression in T24, after treating with 500 nM (light gray bars) and 5 *μ*M (dark gray bars) of four class I HDACi.

We noticed Mocetinostat, Entinostat and CI994 had the highest fold change in the mesenchymal-like cells. We asked whether that was due to different regulation patterns of EMT TFs in cells with different EMT status. In epithelial-like lines PEO1 and OVCA429, Mocetinostat and Entinostat significantly enhanced *ZEB1*, *ZEB2* and *TWIST1* expressions (Table 1). In mesenchymal-like lines SKOV3 and Hey, *ZEB1* and *TWIST1* expressions were reduced by all three HDACi (Table 1). Interestingly, the *SNAI1* and *SNAI2* expressions were consistently induced by HDACi in both epithelial- and mesenchymal-like cell lines (except *SNAI2* in SKOV3) (Table 1). This suggests that the epigenetic regulation of the *SNAI* family is different from the other EMT TFs and might be independent from the intrinsic phenotype. To confirm that our results were not due to different genetic backgrounds of different cell lines, we utilized an isogenic EMT model OVCA429_shLuc (IE) and OVCA429_shGRHL2 (IM)^22^ to test the different effects of HDACi in cells at different EMT states. Consistently, upon the treatment with HDACi, the epithelial-like OVCA429_shLuc had less than twofold increase of *CDH1* expression and significant increases in the expressions of EMT TFs, *SNAI1*, *SNAI2*, *ZEB1* and *ZEB2* (Figures 5a and c); the mesenchymal-like OVCA429_shGRHL2 showed a huge induction of *CDH1* expression but with reciprocal changes between *SNAI1*, *SNAI2* with the *ZEB* family and *TWIST1* (Figures 5b and d). Similar to ovarian cancer cells, the class I HDACi induced significant SNAI family expressions in Mia-Paca2 and T24 cells (Figures 5e and f). These results also indicate that the epigenetic regulations of the EMT TFs are different between the epithelial and mesenchymal cells. Of note, the folds of induction of *CDH1* expression were significantly higher than those of the reduction of EMT TFs. This suggested that the restoration of *CDH1* expression by HDACi was a direct epigenetic regulation to the E-cadherin gene rather an indirect consequence to the downregulation of the EMT TFs.

### Class I HDACi inhibits anchorage independence growth in mesenchymal ovarian and bladder carcinoma cells

**Figure 6:**
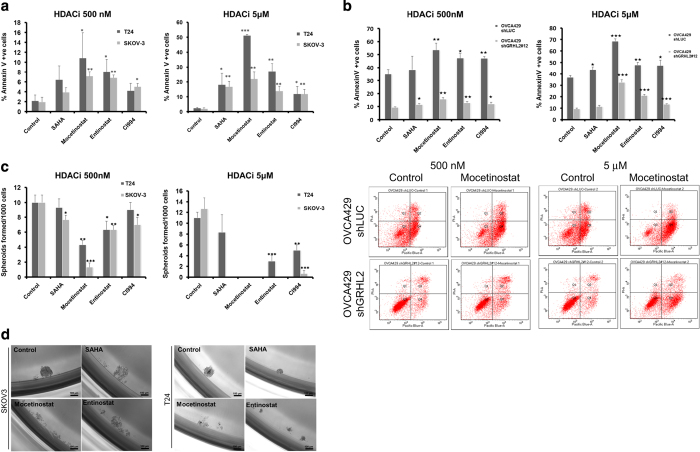
Effects of HDACi on *in vitro* anchorage independence growth. (**a**) Graph represents the percentage of SKOV3 (dark gray) and T24 (light gray) Annexin V-positive (% Annexin V positive) cells (*y*-axis) in suspension culture treated with 500 nM and 5 *μ*M concentration of HDACi (*x*-axis, SAHA, Mocetinostat, Entinostat and CI994) after 48 h of incubation. (**b**) Graph represents the percentage of OVCA429_shLuc (dark gray) and OVCA429_shGRHL2 (light gray) Annexin V-positive (% Annexin V positive) cells (*y*-axis) in suspension culture treated with 500 nM and 5 *μ*M concentration of HDACi (*x*-axis, SAHA, Mocetinostat, Entinostat and CI994) after 48 h of incubation. Representative flow cytometry scatter plots of Annexin V (*x*-axis) and PI (*y*-axis) channels. (**c**) Number of spheroids formed per 1000 cells (*y*-axis) in SKOV3 (dark gray) and T24 cells (light gray) after day 7 treated with 500 nm and 5 *μ*M concentration of HDACi (*x*-axis, SAHA, Mocetinostat, Entinostat and CI994). (**d**) Phase contrast image of SKOV3 and T24 cells treated with 5 *μ*M concentration of HDACi in suspension culture at day 7. All the experiments were performed as three independent experiments, *P<0.05, **P<0.01, ***P<0.001.

EMT reversal and restoration of E-cadherin expression are associated with increased anoikis.^8,9,23^ We subsequently utilized the ovarian carcinoma SKOV3 and bladder carcinoma T24 lines for functional studies to test if these class I HDACi affect the anoikis resistance *in vitro*. The percentage of the Annexin V-positive populations at 48 h in ultra-low attachment cultures was applied as the indication of cells entering apoptosis. The HDACi-treated T24 cells showed significant increase in the cell fractions entering the early anoikis phase (Annexin V^high^/PI^low^ populations; Figure 6a). This anoikis-inducing effect followed dose dependency of HDACi whereas increased percentage of Annexin V-positive cells was found in the 5 *μ*M compared with the 500 nM treated group (Figure 6a; Supplementary Figure 3). In the isogenic EMT model, similar trends were also evident in the mesenchymal OVCA429 shGRHL2 cells that all three class I HDACi significantly enhanced anoikis (Figure 6b). Of note, the epithelial OVCA429 shLUC line consistently showed higher anoikis fraction compared with the mesenchymal OVCA429 shGRHL2 line. In addition, the HDACi-treated cancer cells showed decreased spheroid forming efficiencies. Consistent with the anoikis-inducing effect, Mocetinostat-treated cells showed a significant reduction of spheroid formation at 500 nM in both SKOV3 and T24 (Figure 6c). The effect was even more prominent at 5 *μ*M of Mocetinostat and Entinostat (Figures 6c and d). In conclusion, the HDACi abolished the anchorage independence growth and overcome the anoikis resistance of mesenchymal-like cancer cells while restoring the epithelial differentiation.

## Discussion

Previously, we have proposed two strategies to ascertain the utility of EMT reversal in carcinoma: the first, assessing EMT-related functions, such as the induction of colony compaction; the second, inducing re-differentiation of mesenchymal-like cells by the upregulation of epithelial markers, such as E-cadherin.^9^ In this study, we utilized the second approach and established a 3-phase drug discovery pipeline and identified HDACi to be a potent class of compounds to restore epithelial differentiation.

Increasing reports have demonstrated HDACi to be a promising class of anticancer agents. They act as epigenetic modifiers, by promoting histone hyperacetylation, to influence chromatin remodeling and eventually, gene expression. Being structurally diverse, HDACi is classified into hydroxamic acids, carboxylic acids, benzamides, cyclic peptides and short fatty acids.^24^ Two FDA-approved HDACi, Vorinostat and Romidepsin, are currently in use for cutaneous T-cell lymphoma treatment.^25–28^ Several other HDACi, including Entinostat and Mocetinostat, are under investigation in different phases of clinical trials.^29–31^ The mechanisms of epigenetic modifiers were mainly focused on their anti-proliferative, pro-apoptotic and anti-angiogenic effects.^32,33^ However, the role of HDACi in EMT remains unclear. In fact, contradicting data, either promoting EMT or inhibiting EMT by HDACi, have been reported.

Vorinostat or Trichostatin A treatment was found to induce EMT through upregulation of *ZEB1*, *ZEB2*, *SNAI1* and *SNAI2* expression, with associated increased expression of mesenchymal markers, N-cadherin and vimentin, in prostate cancer cells.^34^ Similar results were obtained in human nasopharyngeal carcinoma cells (CNE2), colon cancer cells (LoVo) and liver carcinoma cells (HepG2). These cancer cells demonstrated increased *SNAI1* expression and metastasis, coupled with a fibroblast-like morphology of mesenchymal cells, after Vorinostat or Sodium butyrate (NaB) treatment.^35^ On the contrary, treatment with either Vorinostat or Trichostatin A, squamous cell carcinoma of the head and neck (SCCHN) cells, induced E-cadherin expression and suppressed TGF*β* expression, an indication of EMT inhibition.^36^ Recently, several epigenetic modifiers have indeed been reported to reverse the aggressiveness associated with EMT in solid tumors.^37–40^

Meidhof *et al*.^39^ reported that Mocetinostat reversed the gemcitabine resistance in pancreatic cancer via suppressing the EMT TF *ZEB1*. However, based on our results, Mocetinostat increased *ZEB1* expression significantly in epithelial-like lines PEO1 and OVCA429, correlating with increased resistance to HDACi treatment. On the other hand, mesenchymal-like cell lines, SKOV3 and Hey, demonstrated suppressed *ZEB1* expression after Mocetinostat treatment, correlating with increased sensitivity to HDACi treatment. Interestingly, epithelial-like ovarian cancer cells had been shown to be more resistant to cisplatin than mesenchymal-like ovarian cancer cells.^41^ Thus, the intrinsic EMT states of cancer cells might dictate their difference in drug sensitivity to both HDACi and conventional chemotherapeutics. Indeed, in our hands, prior short-term exposure to HDACi did not sensitize SKOV3 cells to paclitaxel or cisplatin (Supplementary Figure 4). Therefore, the combinatory usage of HDACi with other cytotoxic agents would need to be carefully investigated for the optimized dosing schedules.

We also observed that MDA-MB-231 has the highest EpIC-50 among all tested cell lines. This is supported by the work done by Rhodes *et al.*^38^ in which Vorinostat-treated MDA-MB-231 did not change morphologically and had no significant changes in EMT-related gene expression. Similar findings were reported by El-Kenawi *et al*.^42^ that combination of Vorinostat and cisplatin was unable to trigger apoptotic responses in MDA-MB-231. Drug insensitivity in MDA-MB-231 may be due to the presence of tumor-initiating cells.^40^ Schech *et al.* suggested Entinostat treatment could overcome tumor-initiating cells and to curb tumor development and lung metastasis in MDA-MB-231 *in vivo* models. Even though we did not test Entinostat on MDA-MB-231, it suggests that HDACi not just has the potential in curbing EMT, but also to act as a corrective measure to overcome drug insensitivity in cancer cells. Among the HDACi tested, we found that Mocetinostat induced the highest fraction of Annexin V-positive cells compared with Entinostat and Vorinostat. This trend is also negatively correlated with the EpIC-50 result, suggesting that HDACi with the highest potency to restore E-cadherin expression demonstrated by having a lower EpIC-50 value would also show an enhanced potency in inducing anoikis.

From the EpI-50 results, the HDACi with high potency in inducing the epithelial gene expressions are all class I HDACi. Mocetinostat has the specificity against HDAC1, 2, 3, 11; Entinostat and CI994 are both specific to HDAC1 and 3. HDAC1, HDAC2 and HDAC3 have all been shown to regulate the expression of epithelial genes and EMT. The suppression of E-cadherin is mediated by a SNAIL/HDAC1/HDAC2 repressor complex.^43,44^ HDAC3, by interacting with hypoxia-induced WDR5, serves as an essential corepressor to repress epithelial gene expression.^45^ Intriguingly, these HDACi all significantly induce *SNAI1* expressions in both epithelial- and mesenchymal-like lines. Being a strong transcriptional repressor to *CDH1*, the *SNAI1* induced by HDACi does not decrease the *CDH1* expression in these lines. On the contrary, the *CDH1* expression is greatly enhanced in the mesenchymal-like lines. These data suggested that class I HDACs are pivotal in the downregulation of epithelial genes during EMT. Therefore, the class I HDACs, even at the presence of a strong EMT TF such as SNAI1, are a very promising class of target for restoring epithelial differentiation.

In addition to using the E-cadherin promoter as the readout, we also show that promoters of other epithelial genes, such as *ERBB3*, can also be utilized for EpI screen. ErbB3 belongs to the epidermal growth factor receptor family. It has emerged as a promising therapeutic target in cancer by using monoclonal antibodies.^46^ Vorinostat was reported to revert the mesenchymal phenotype and enhance the antitumor effect of gefitinib by inducing both E-cadherin and ErbB3 expressions.^47^ In our hands, the class I HDACi shows even lower EpIC-50 in inducing the *ERBB3* promoter activities than that in *CDH1*. This suggests that restoring ErbB3 expressions might be easier to achieve to overcome the epigenetic barriers. Our group has previously shown that *ERBB3* is a direct transcriptional target of an epithelial-specific TF, GRHL2^22^, and the ErbB3 expression and its phosphorylation are significantly decreased along an EMT spectrum.^8^ Therefore, sensitizing the mesenchymal-like tumors expressing low ErbB3 with HDACi would be a promising strategy. Recently, synergistic antitumor activity of HDACi and anti-ErbB3 antibody has been reported in NSCLC primary cultures.^48^

In summary, we report here a drug screening pipeline for the discovery of classes of compounds to restore epithelial differentiation. We found that HDAC inhibitors achieved the EMT reversal and epithelial differentiation effect by restoring E-cadherin and ErbB3 expressions in a panel of cancer cells with an intermediate EMT state. The identified HDACi also showed functional relevance in reversing anoikis resistance and abolishing spheroid formation. Future combination of HDACi with agents targeting ErbB3 signaling is promising.

## Materials and methods

### *In vitro* culture of cancer cell line

The human OC cell lines, SKOV3 (ATCC, Manassas, VA, USA) and OVCA429 (from Dr. Noriomi Matsumura, Kyoto University), and non-OC cell lines, MDA-MB-231, MIA-PaCa2, and T24 (ATCC), were maintained in complete high-glucose Dulbecco’s modified Eagle’s medium (Biowest SAS, Nuaillé, France), supplemented with 10% (v/v) fetal bovine serum (Biowest SAS, Nuaillé, France). Hey and PEO1 (both from Dr. Noriomi Matsumura, Kyoto University) were maintained in RPMI11640 (Biowest SAS, Nuaillé, France), supplemented with 10% (v/v) fetal bovine serum (Biowest SAS, Nuaillé, France) and 2 mM NaPy (Sigma-Aldrich, St. Louis, MO, USA). All cells were cultured at 37 °C in a humidified atmosphere containing 5% CO_2_ and 95% air.

### Compound library screens

A FDA-approved 853 drug library was purchased from Selleckchem (Houston, TX, USA) and used in Phase I EpI screen. Compounds, in the form of 10 mM in DMSO, were diluted to 1 mM and kept at −20 °C, to be used on day 3 drug treatment. Class I HDACi (10 mM in DMSO), used in Phase 2 EpI screen, were obtained from Inhibitor Library (Selleckchem, Houston, TX, USA). Selected class I HDACi—Vorinostat, Mocetinostat, Entinostat and CI994—were purchased from Selleckchem in the form of powder and reconstituted in DMSO to a concentration of 10 mM and kept at −20 °C.

Cells of interest were plated at 40–50% confluence per well, in complete media, into 96-well plates (no. 655094, Greiner). After 24 h, cells were transfected with 100 ng of pGL3-Ecad or vector control and 1.5 ng of pRL-CMV Renilla vector per well, using X-tremeGENE HP DNA transfection reagent (no. 6366236001, Roche, Mannheim, Germany) at 2 : 1 HP : DNA ratio. On day 3, cells were treated with 5 *μ*M of drug (Selleckchem, final concentration) or a similar volume of DMSO (for control conditions, final concentrations: 0.05% v/v). After 24 h of drug treatment, Dual-Glo Luciferase Assay was performed according to the manufacturer’s protocol (no. E2940, Promega, Madison, WI, USA).

### EpIC-50 determination

All cells were plated and transfected as described above. On day 3, SKOV3 were treated with Vorinostat or CI994 (both at 1 mM, 200, 40, 8, 1.6 *μ*M, 320, 64, and 12.8 nM), Mocetinostat (2, 1 *μ*M, 800, 600, 400, 200, 100 and 50 nM) or Entinostat (8, 4, 2, 1 *μ*M, 500, 250, 125 and 62.5 nM) for 24 h. MDA-MB231 was treated with Vorinostat of concentrations, 1 mM, 200, 40, 8, 1.6 *μ*M, 320, 64, and 12.8 nM) for 24 h. MIA-PaCa2 was treated with Vorinostat or CI994 (both at 1 mM, 200, 40, 8, 1.6 *μ*M, 320, 64, and 12.8 nM), Mocetinostat (64, 32, 16, 8, 4 *μ*M, 800, 400, and 200 nM), or Entinostat (400, 200, 100, 50, 25 *μ*M, 12.5 nM, 6.25, and 3.125 *μ*M) for 24 h. T24 was treated with Vorinostat or CI994 (both at 1 mM, 200, 40, 8, 1.6 *μ*M, 320, 64, and 12.8 nM), Mocetinostat (16, 8, 4, 2, 1 *μ*M, 200, 100, and 50 nM), or Entinostat (8, 4, 2, 1 *μ*M, 500, 250, 125, and 62.5 nM) for 24 h.

All drug treatments were at final concentration. Ecad promoter activity of all cell lines were measured using Dual-Glo Luciferase Assay. EpIC-50 was generated by curve fitting using three-parameter analysis.

### Nucleic acid isolation

RNA was isolated based on the protocols described in the miRNeasy Kit (#217004, Qiagen, Valencia, CA, USA). Briefly, cells were lysed in TRIzol Reagent (no. 15596026, Life Technologies, Carlsbad, CA, USA) (800 *μ*l/10 cm^2^ culture surface area), and the lysate vigorously mixed with 160 *μ*l of chloroform before separating the aqueous phase via centrifugation (12 000×*g*, 15 min, 4 °C). RNA was precipitated with 1.5× volume of absolute ethanol (of aqueous phase) and purified using the spin columns (provided in the kit). Isolated RNA was subsequently re-suspended in nuclease-free water (provided in the kit). RNA concentration was determined using the NanoDrop 1000 Spectrophotometer (Thermo Fischer Scientific, Waltham, MA, USA).

### Quantitative PCR on EMT genes

PEO1, OVCA429, SKOV3, Hey, OVCA429 shLuc, OVCA429 shGRHL2 and MIA-PACA2 were grown in 60-mm tissue culture plates (Corning Inc., Corning, NY, USA) until 90% confluence. Each cell line was grown in duplicate. RNA was extracted and purified as described above. RNA of 500 ng was reverse transcribed into cDNA using RT^2^ First Strand Synthesis Kit (Qiagen, Germantown, MD, USA) and subjected to real-time QPCR analysis of *SNAI1*, *SNAI2*, *CDH1*, *TWIST1, GRHL2, ZEB1* and *ZEB2* using RT^2^ qPCR SYBR/ ROX Master Mix and equal volumes of mixtures. Five housekeeping genes (HKGs), *ACTB*, *B2M*, *GAPDH*, *HPRT1* and *RPL13A*, were used as assay controls. Data in the form of threshold cycle numbers (*C*_t_) were uploaded to the online data analysis portal (http://pcrdataanalysis.sabiosciences.com/pcr/arrayanalysis.php) to calculate the delta-C_t_ (Δ*C*_t_). *C*_t_ was determined through the SDS (version 2.3) software (Applied Biosystems, Carlsbad, CA, USA) by setting the baseline between cycle 2 of the run (total run: 40 cycles) and two cycles before the start of the first log-phase amplification. The threshold was set by positioning the limit to the lower one-third of the earliest amplification. Δ*C*_t_ was calculated by the respective formulae below:

$$\Delta C_{t}=C_{t}\left(\mathrm{GOI}\right)-C_{t}\left(\mathrm{HKG}\right)$$

where by: *C*_t_ (gene of interest, GOI): *C*_t_ value of the respective GOI, *C*_t_ (HKG): average *C*_t_ values of the five HKGs used in the assay.

### Anoikis assay

SKOV3 and T24 cells were seeded in 10 cm ultra-low attachment dish and treated with two different concentrations of 500 nM and 5 *μ*M of HDACi (SAHA, Mocetinostat, Entinostat and CI994) at the time of seeding. After 48 h incubation, the cells were collected and trypsinized into single cell suspensions. The cells were then washed twice with PBS and counted using a hemocytometer. Equal numbers of cells were taken from control and HDACi, then transferred into a 15-ml Falcon tube (BD Biosciences, San Jose, CA, USA). The cells were re-suspended in 100 *μ*l of 1× Annexin V binding buffer. Annexin-Pacific Blue conjugate (5 *μ*l; Sigma-Aldrich) and 1 *μ*l of 100 *μ*g/ml Propidium Iodide (Sigma-Aldrich) working solution were added and incubated for 15 min at room temperature. After the incubation period, 400 *μ*l of 1× annexin-binding buffer were mixed gently and kept the samples on ice. Data were acquired using a BD LSRII flow cytometer (BD Biosciences). A minimum of three independent experiments was performed to study Anoikis assay.

### Spheroid forming assay

SKOV3 and T24 cells were cultured in 2D culture dish up to 70% of confluency. The cells were trypsinized and counted using a hemocytometer. In each well of 96-well ultra-low attachment plates, 200 cells were seeded and treated with HDACi (SAHA, Mocetinostat, Entinostat and CI994) with two different concentrations of 500 nM and 5 *μ*M at the time of seeding. The cells were incubated for 7 days and spheroids were manually counted at day 7. Each HDACi drug study has been performed with three independent experiments.

## Figures and Tables

**Table 1:** Summary of the effect of HDACi on EMT transcription factors expression in epithelial- and mesenchymal-like cells

	*PEO1*		*OVCA429*		*SKOV3*		*HEY*	
	*Fold change±S.E.M.*	P*-value*	*Fold change±S.E.M.*	P*-value*	*Fold change±S.E.M.*	P*-value*	*Fold change±S.E.M.*	P-*value*
*Mocetinostat (5 μM)*								
SNAI1	17.837±0.32	0.06	46.32±2.20	0.01	11.80±0.01	6.85E-05	71.84±0.5	0.001
SNAI2	7.4±0.79	0.03	2.83±0.17	0.02	0.72±0.07	0.10	8.46±0.73	0.03
ZEB1	12.79±0.28	0.006	12.31±0.18	0.001	0.75±0.06	0.05	0.36±0.07	0.006
ZEB2	20.70±4.88	0.07	6.02±0.69	0.04	1.54±0.25	0.13	0.35±0.05	0.02
TWIST1	3.07±0.01	0.0002	2.73±0.10	0.016	0.72±0.03	0.01	0.78±0.07	0.08
GRHL2	1.28±0.09	0.09	0.36±0.004	0.037	1.93±0.6	0.19	1.43±0.01	0.01
								
*Entinostat (5 μM)*								
SNAI1	8.52±0.01	1.3E-05	11.9±0.25	0.005	5.47±0.03	0.0001	31.71±0.39	0.002
SNAI2	5.01±0.23	0.01	1.36±0.02	0.01	0.66±0.019	0.1	5.96±0.37	0.02
ZEB1	9.63±0.15	0.002	5.18±0.31	0.018	0.75±0.03	0.01	0.34±0.01	0.001
ZEB2	14.68±2.78	0.06	2.26±0.28	0.05	1.54±0.04	0.02	0.32±0.01	0.04
TWIST1	2.54±0.05	0.009	1.38±0.05	0.03	0.83±0.08	0.14	0.61±0.003	0.047
GRHL2	1.21±0.01	0.008	0.44±0.009	0.04	0.72±0.01	0.28	1.00±0.23	0.49
								
*CI994 (5 μM)*								
SNAI1	2.13±0.03	0.001	5.72±0.11	0.002	2.78±0.11	0.01	18.59±1.89	0.03
SNAI2	1.14±0.12	0.22	1.16±0.03	0.03	0.67±0.01	0.1	3.82±0.20	0.02
ZEB1	2.48±0.1	0.008	2.49±0.24	0.04	0.82±0.02	0.02	0.58±0.02	0.002
ZEB2	4.35±0.80	0.07	1.13±0.25	0.35	1.76±0.003	0.0001	0.68±0.06	0.06
TWIST1	1.04±0.02	0.17	1.09±0.09	0.04	0.87±0.02	0.02	0.70±0.01	0.05
GRHL2	1.03±0.04	0.31	0.76±0.02	0.08	1.47±0.91	0.38	1.08±0.21	0.08

## Notes

Supplementary Figure

Supplementary Informations
