# Supplementary material for: An epithelial marker promoter induction screen identifies histone deacetylase inhibitors to restore epithelial differentiation and abolishes anchorage independence growth in cancers
Source: Cell Death Discov. 2016 Jun 13;2:16041–. doi: 10.1038/cddiscovery.2016.41 (PMC4979427; doi:10.1038/cddiscovery.2016.41)
Supplement: Supplementary Figure [file cddiscovery201641-s1.ppt]

## Slide 1
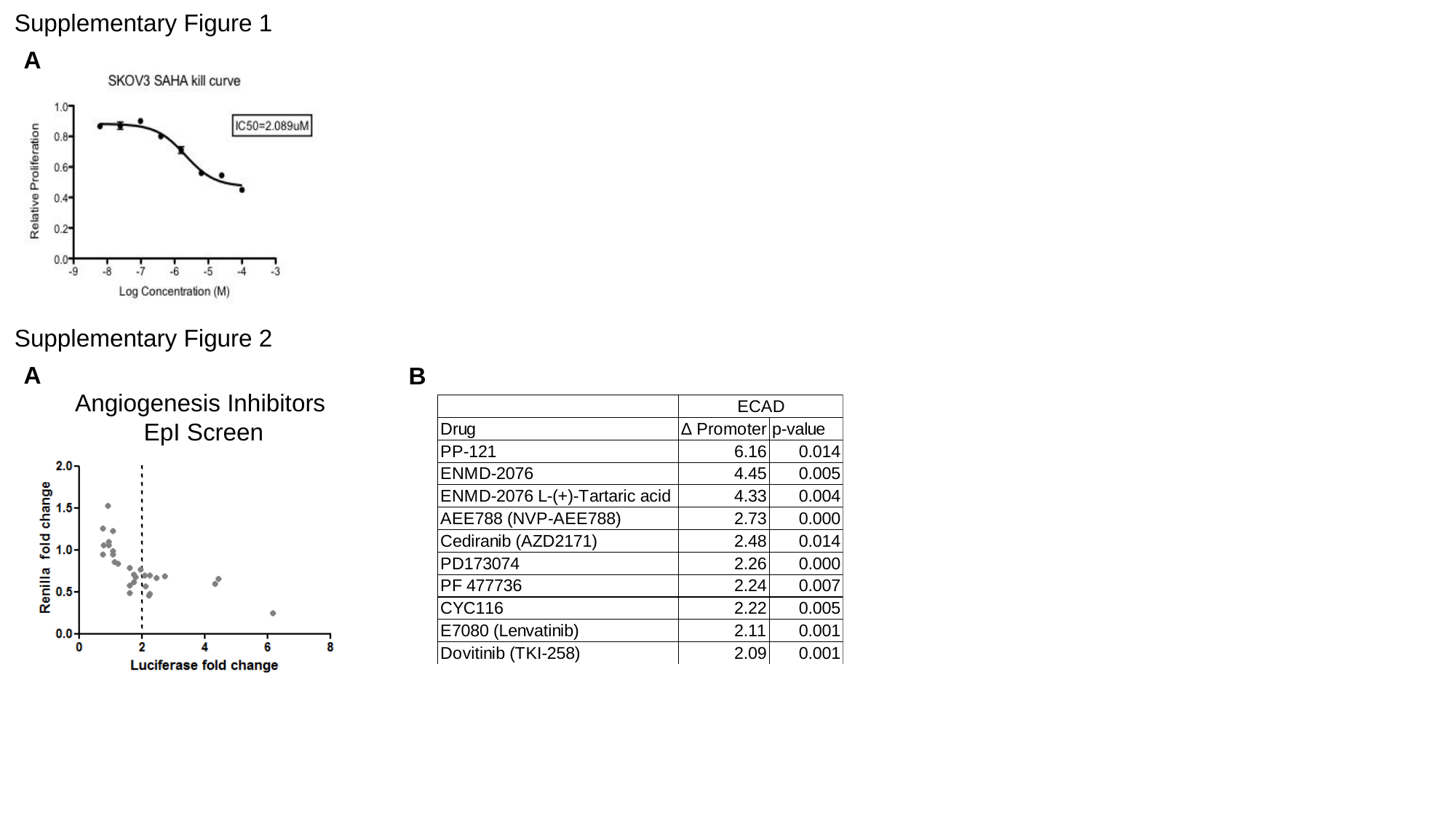

Supplementary Figure 1
A
Supplementary Figure 2
A
B
Angiogenesis Inhibitors
EpI Screen

## Slide 2
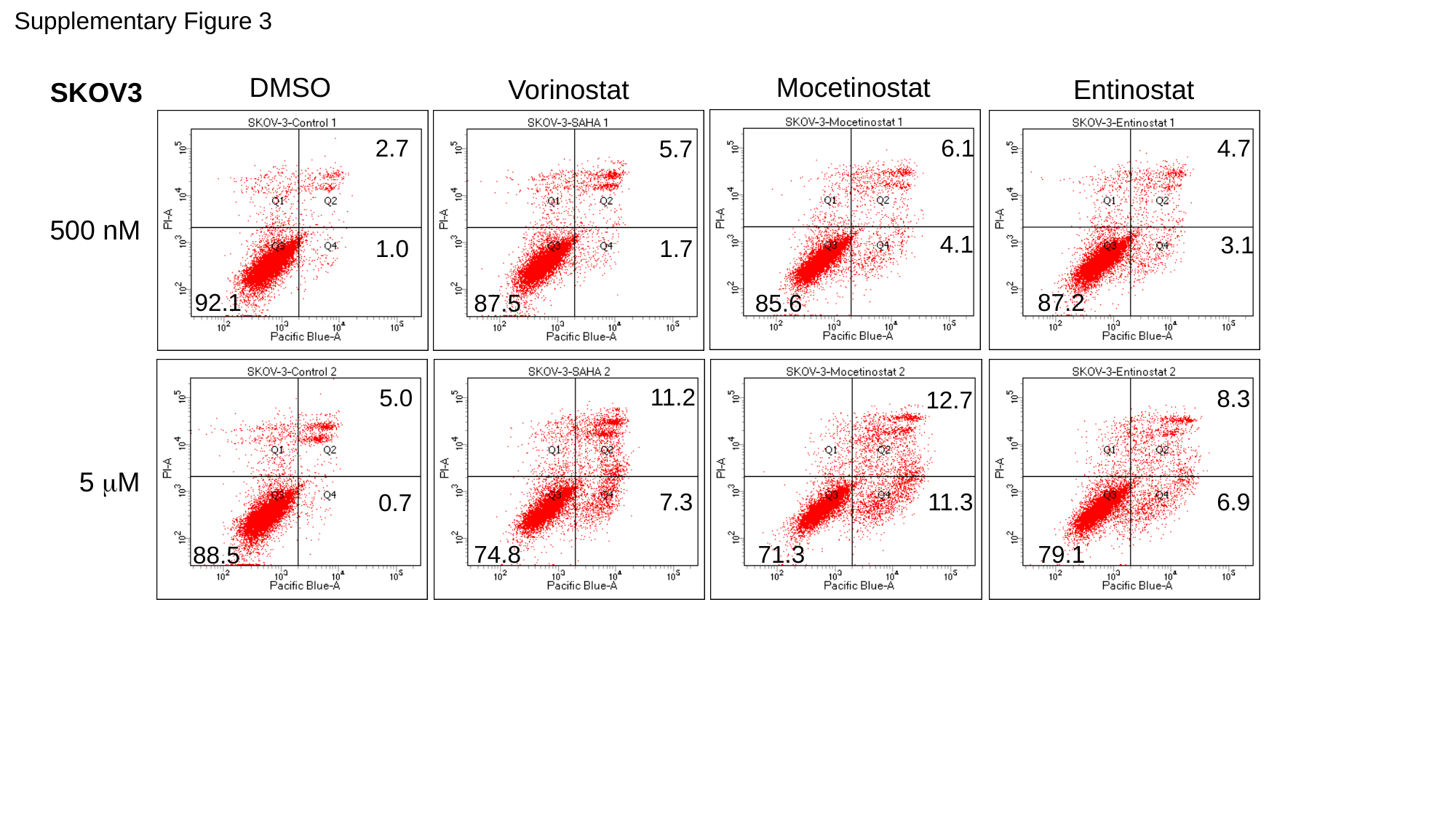

Supplementary Figure 3
DMSO
Mocetinostat
Vorinostat
Entinostat
SKOV3
2.7
6.1
4.7
5.7
500 nM
4.1
3.1
1.0
1.7
92.1
87.2
87.5
85.6
11.2
5.0
8.3
12.7
5 μM
11.3
6.9
7.3
0.7
74.8
71.3
79.1
88.5

## Slide 3
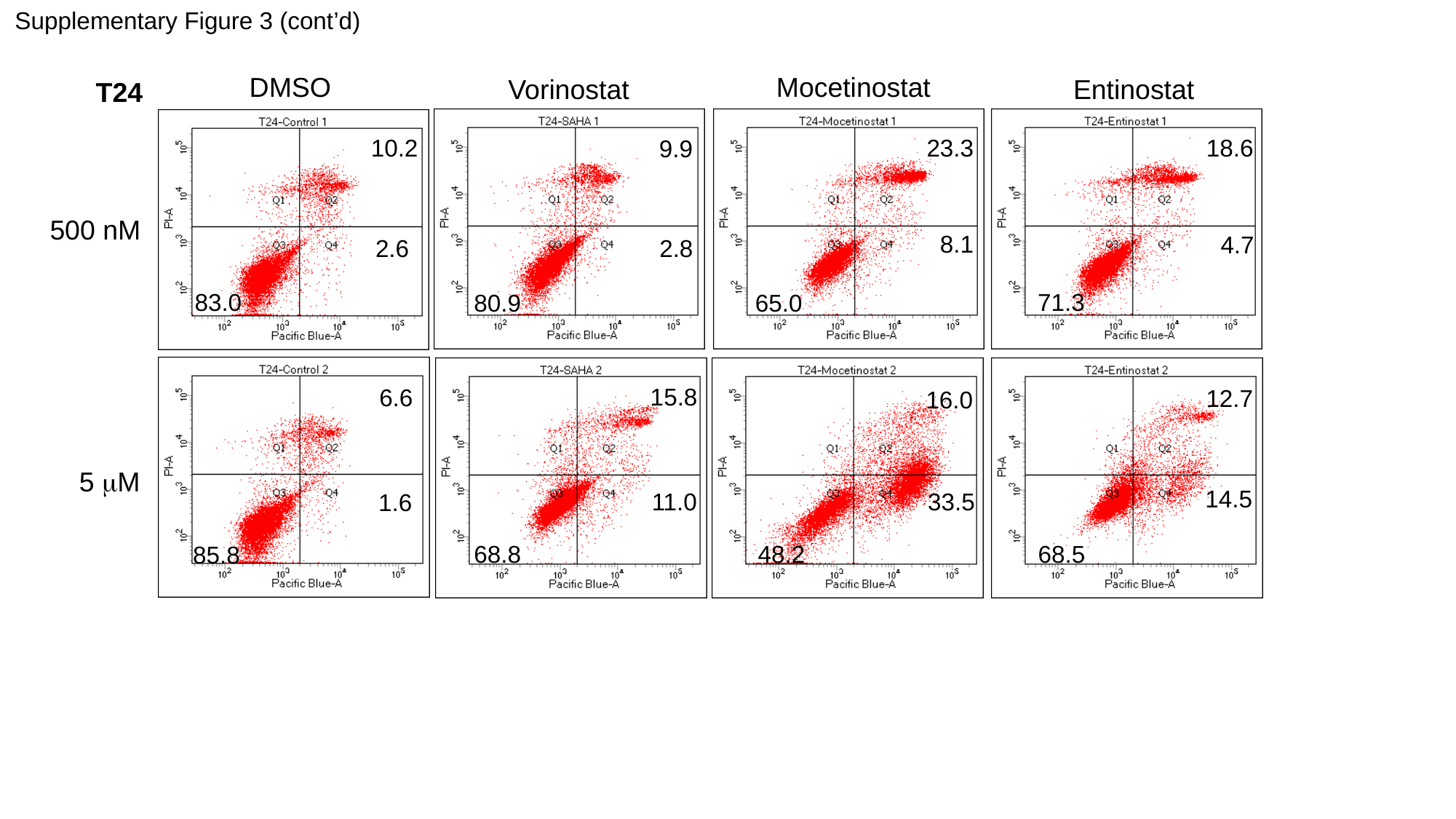

Supplementary Figure 3 (cont’d)
DMSO
Mocetinostat
Vorinostat
Entinostat
T24
10.2
23.3
18.6
9.9
500 nM
8.1
4.7
2.6
2.8
83.0
71.3
80.9
65.0
15.8
6.6
12.7
16.0
5 μM
14.5
33.5
11.0
1.6
68.8
48.2
68.5
85.8

## Slide 4
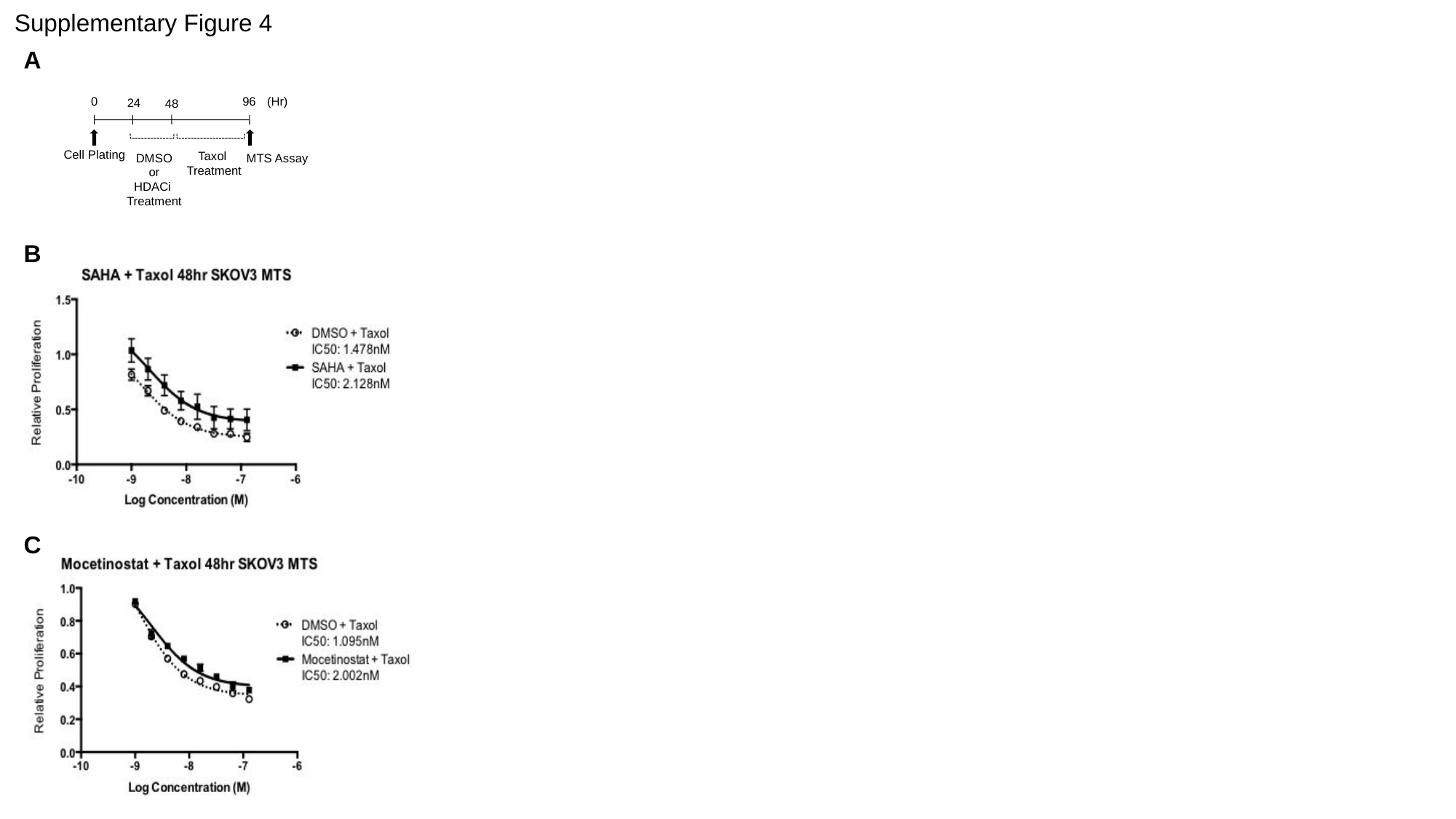

Supplementary Figure 4
A
0
96
(Hr)
24
48
Cell Plating
Taxol
Treatment
DMSO
or
HDACi
Treatment
MTS Assay
B
C
