# Supplementary material for: An epithelial marker promoter induction screen identifies histone deacetylase inhibitors to restore epithelial differentiation and abolishes anchorage independence growth in cancers
Source: Cell Death Discov. 2016 Jun 13;2:16041–. doi: 10.1038/cddiscovery.2016.41 (PMC4979427; doi:10.1038/cddiscovery.2016.41)
Supplement: Supplementary Informations [file cddiscovery201641-s2.doc]

**SUPPLEMENTARY FIGURE LEGENDS**

**Supplementary Figure 1. MTS cell proliferation assay for Vorinostat (SAHA).** Dose-response curve indicates IC_50_ of Vorinostat (SAHA) in SKOV3. *y*-axis represents the relative cell proliferation of SKOV3 and *x*-axis indicates various concentrations of Vorinostat. IC_50_ values were measured using CellTiter 96® AQueous One Solution Cell Proliferation Assay (MTS), and generated by curve fitting using three-parameter analysis.

**Supplementary Figure 2. Effect of angiogenesis inhibitors on E-cadherin promoter activity.** (A) Dot plot summarizes the results of Phase 1 EpI Screen for angiogenesis inhibitors. *y*-axis indicates cell viability and *x*-axis represents induction of Ecad promoter activity. Ten compounds were identified with more than 2-fold Ecad promoter activity. (B) Table summarizes the fold-change of Ecad promoter activity induction by angiogenesis inhibitors. *P*-value indicates the change in Ecad promoter activity analysis using paired t-test for comparison between drug-treated and DMSO-treated groups.

**Supplementary Figure 3.** **Effects of HDACi on *in vitro* anchorage independence growth.** Representative flow cytometry scatter plots of AnnexinV (*x*-axis) and PI (*y*-axis) channels for SKOV3 and T24.

**Supplementary Figure 4. Effect of HDACi on cell sensitization to paclitaxel.** (A) Schematic illustration of the experimental design. Cells were treated, with or without HDACi (SAHA, Mocetinostat), 24 h prior to palitaxel treatment and relative cell proliferation was assessed thereafter. Dose-response curves indicate IC_50_ for (B) Vorinostat-paclitaxel treatment, and (C) Mocetinostat-paclitaxel treatment in SKOV3.
